# Whole blood fatty acid profile of young subjects and adherence to the Mediterranean diet: an observational cohort study

**DOI:** 10.1186/s12944-022-01633-x

**Published:** 2022-02-17

**Authors:** Marie-Louise Syrén, Stefano Turolo, Erika Adalgisa de Marco, Valentina De Cosmi, Patrizia Risé, Franca Marangoni, Dario Guido Minoli, Gianantonio Manzoni, Carlo Agostoni

**Affiliations:** 1 Department of Clinical Sciences and Community Health, University of Milan, Via Commenda 9, 20122 Milan, Italy; 2 Pediatric Nephrology, Dialysis and Transplant unit, Fondazione IRCCS Ca’ Granda Ospedale Maggiore Policlinico, Via Commenda 9, 20122 Milan, Italy; 3 Pediatric Urology unit, Fondazione IRCCS Ca’ Granda Ospedale Maggiore Policlinico, Via Commenda 9, 20122 Milan, Italy; 4 Pediatric Intermediate Care unit, Fondazione IRCCS Ca’ Granda Ospedale Maggiore Policlinico, Via Commenda 9, 20122 Milan, Italy; 5 Department of Pharmaceutical Sciences, University of Milan, Via Balzaretti 13, 20133 Milan, Italy; 6 NFI, Nutrition Foundation of Italy, Viale Tunisia 38, 20124 Milan, Milan Italy

**Keywords:** Childhood, Adolescence, Whole blood, Fatty acids, Polyunsaturated fatty acids (PUFA), n3-PUFA, n6-PUFA, Mediterranean diet, Mediterranean diet quality index for children (KIDMED)

## Abstract

**Background:**

Relatively little is known about the physiological whole blood fatty acid composition in young people. Likewise, few studies have addressed the question of correlations between Mediterranean diet (MedDiet) adherence and blood fatty acids in childhood.

**Methods:**

The fatty acid profile in whole blood from subjects, 46 days-19 years old (*n* = 152), without acute, chronic, or inflammatory diseases was analysed by gas chromatography. Dietary data was extracted from a 24-h recall in a subgroup of subjects (*n* = 60) into a modified Diet Quality Index for Children (KIDMED) questionnaire to evaluate MedDiet adherence. The cohort was divided into three age groups: < 2, 2- < 10, and 10–19 years. Kruskal-Wallis test and Bonferroni post hoc test were used to check for age group fatty acid differences. For correlations, Spearman’s correlation coefficient and partial Spearman’s correlation coefficient were used.

**Results:**

Linoleic acid, EPA, DHA, palmitic acid, and total saturated fatty acids were stable over age groups. Dihomo-gamma-linolenic acid (DGLA), arachidonic acid (AA), total polyunsaturated FAs (PUFA), and total omega-6 PUFA increased from age group < 2 years; alpha-linolenic acid, total omega-3 PUFA, oleic acid, and total monounsaturated FAs decreased. Adherence to the MedDiet was at low-medium level in 91.7% of the subjects. In the age group 2- < 10 yrs., the degree of adherence correlated positively with total MUFA and PUFA balance, negatively with total PUFA, total n6-PUFA, AA/DHA, AA/EPA, and n6/n3. Age did not influence the correlations as to PUFA balance and AA/EPA.

**Conclusions:**

Increased FA proportions with age were seen in the n6-series of PUFA. The n3-FA species decreased or were stable. The vast majority of the subjects with dietary data, 92%, obtained a KIDMED score indicative of low-medium adherence to the MedDiet. The score correlated negatively with various n6-species, i.e. the MedDiet suppressed circulating n6-PUFA. Whole blood may be used to investigate FAs and MedDiet adherence correlations which may be applied in the study of health issues in childhood.

## Background

Fatty acids (FAs) perform a multitude of functions in the body such as signalling in cells that affect transcription, act as precursors for metabolites with biological activity, constitute essential components of membranes that influence the physicochemical properties, among others [1]. The composition of blood FAs is the result of food intake, endogenous synthesis, and metabolic requests [2]. Life-related events (like pregnancy) and lifestyle factors (such as smoking) [3] as well as different disorders and diseases [4–7] affect the baseline FA profile. Knowledge of FA status may be especially crucial in children that for development and growth require metabolic substrates. The significance of FAs is reflected, for instance, in the role of the long-chained polyunsaturated FAs (PUFA) for brain maturation and function [8]. Likewise, saturated and monounsaturated FAs (SFA and MUFA, respectively) are essential with regard to preterm and term life [9–12]. Linoleic acid (LA), parent FA of the n-6 series of the PUFA family, undergoes enzymatic transformation through elongation and desaturation to form long-chain PUFA (LCPUFA), such as dihomo-gamma-linolenic acid (DGLA) and arachidonic acid (AA). Alpha-linolenic acid (ALA), the parent FA of the n-3 series, uses the same set of enzymes as LA to be converted into the n3-LCPUFA eicosapentaenoic acid (EPA) and docosahexaenoic acid (DHA). The LCPUFA are matter of extensive investigations for possible roles in health-related conditions, however relatively few studies analysed the FA composition in whole blood in childhood [13–15]. The human body lacks the ability to synthesize LA and ALA, i.e. they are essential FAs so humans must rely on the presence of pre-formed LA and ALA in the diet.

The traditional eating habits in the Mediterranean area, i.e., the Mediterranean diet (MedDiet), are considered a salutary diet as reports showed it reduced total mortality and death caused by coronary heart disease and cancer due to the preferred intake of olive oil, vegetables, fruits, cereals, nuts, legumes, and fish, over saturated fats, meat and poultry [16]. Adherence to the MedDiet and its relation to health outcomes in children and adolescents in Spain, Greece, Cyprus, and Italy was reviewed recently [17]. Despite the presumed healthy effects of the MedDiet, the authors stressed that individuals living in countries with healthy dietary traditions are among those with high(est) prevalence of overweight and obesity [17]. The Mediterranean Diet Quality Index for children (KIDMED) questionnaire was developed previously to measure adherence to the MedDiet by young subjects [18]. To the best of our knowledge, the correlation between adherence to the MedDiet using the KIDMED test and the FA profile in whole blood has not been evaluated in children.

The aim of the present study was to determine FA composition in spotted whole blood in a cohort of young subjects without acute, chronic, or inflammatory disease, to estimate adherence to the MedDiet, and to search for possible correlations between the KIDMED adherence score and blood FA composition.

## Methods

### Study population

**Fig. 1:**
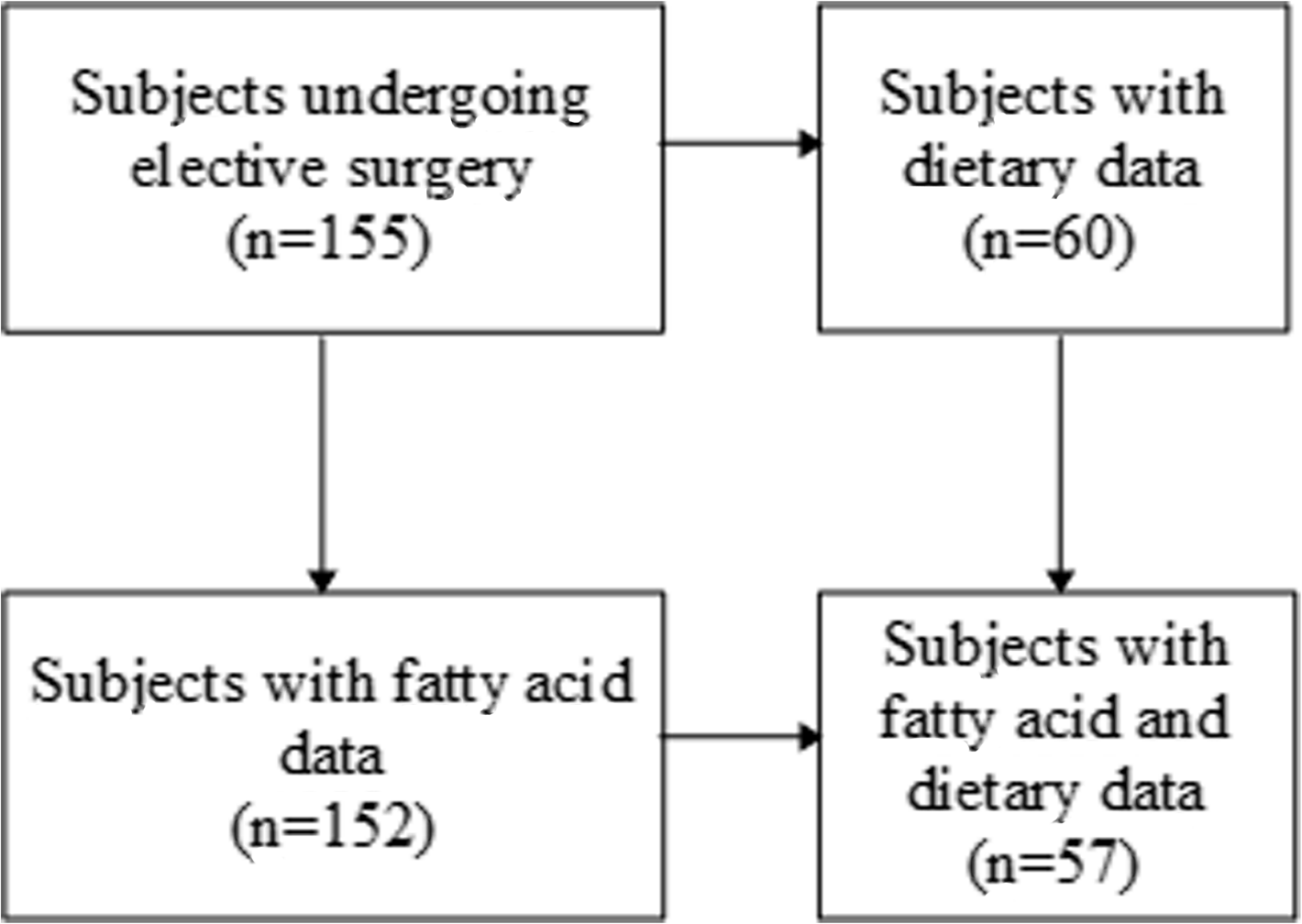
Flow chart of the cohort

The cohort consisted of 155 subjects from the Paediatric Urology unit of Fondazione IRCCS Ca′ Granda Ospedale Maggiore Policlinico in Milan, Italy (Fig. 1). They were referred to the clinic for corrective elective surgery and did not show evidence of acute, chronic, or inflammatory disease.

### Blood sample preparation

Venous blood was collected on Whatman 903 chromatography paper (Sigma-Aldrich, Steinheim, Germany) prepared with the antioxidant butylated hydroxytoluene (BHT) (Fluka, Charlotte, North Carolina, USA) as reported [19]. The chromatography paper with collected blood was kept in a sealed envelope with desiccant at 4° before delivery to the laboratory pending analysis at − 20 °C.

### Fatty acids

Select FA are shown, for their abundance and/or biological activity: palmitic acid (C16:0) and stearic acid (C18:0) of the SFA; oleic acid (C18:1n9) of the MUFA; ALA (C18:3n3), EPA (C20:5n3), and DHA (C22:6n3) of the n-3 PUFA; LA (C18:2n6), DGLA (C20:3n6), and AA (C20:4n6) of the n-6 PUFA. Several indices were calculated: the inflammatory state ratios AA/EPA, AA/DHA, and n6/n3 [20], the PUFA balance marker (%EPA + %DHA)/total PUFA × 100, and estimated enzymatic activity from product and substrate ratios: SCD(18) (C18:1n9/C18:0), FADS2 (DGLA/LA, and FADS1 (AA/DGLA).

### FA analysis

The blood spot was treated as previously described [19]. In brief, blood spot FAs were transmethylated with 3 N Methanol-HCL (Supelco, Steinheim, Germany) for 1 h at 90 °C. The fatty acid methyl esters (FAMEs) were extracted with hexane (Honeywell, Charlotte, North Carolina, USA) and analysed by gas chromatography Nexis GC-2030 (Shimadzu GC-2030, Kyoto, Japan) equipped with a capillary column FAMEwax 30 m (RESTEK, Bellefonte, Pennsylvania, USA), using helium as carrier gas. The PTV injector temperature was 260 °C, that of the FID detector 250 °C. A temperature program was used for analysis. The Labsolutions software (Shimadzu, Kyoto, Japan) was used for elaboration of the chromatogram. FAs were identified using the retention times of reference standards and expressed as % of total FAs.

### Dietary data

Out of 155 subjects admitted to the pediatric urology unit, dietary data was available for 60 (Fig. 1). A 24-h dietary recall was completed by the subjects or their parents/guardians in an interview that lasted about 30 min. Consumed foods were traced and data extracted to a convalidated modified KIDMED questionnaire [21]. The score obtained reflects adherence to the MedDiet in that, a low score indicates low adherence to the MedDiet, a high score indicates high adherence [18, 21]. Scores ≤2*,* 3–6, and ≥ 7 were considered low, intermediate, and high level, respectively. Focus was put especially on children in preschool/primary school ages.

### Statistical analysis

Open Office spread sheets (Microsoft) and SPSS version 21 (IBM) were used for descriptive statistics and/or the Kruskal-Wallis test, Bonferroni post hoc test, Spearman’s correlation coefficient, and partial Spearman’s correlation coefficient, as appropriate. An alpha value of 0.05 (two-tailed) was chosen as probability of type I error, i.e. 95% confidence for significant results and a beta value of 0.20 as acceptable probability of type II error, i.e. a statistical power of 80%. Data are reported as mean and SD or 95% confidence interval (CI).

## Results

### The cohort

Figure 1 shows the flow chart of the cohort study. The cohort consisted of 155 subjects, of which 72% were males, 28% females. They ranged in age from 46 days to 19 years of age. The subjects were divided into three groups according to age: < 2 years (‘small children’), 2- < 10 years (‘school age’ children) and subjects 10 years and older (‘adolescent’ group). Table 1 shows the distributions of age and anthropometric parameters. The cohort showed BMI and z-BMI in the lower range in all age groups.

**Table 1:** Age and anthropometric characteristics of the subjects

Subjects	Age (years)		Height/length (m)		Weight (kg)		BMI (kg/m^2^)		z-BMI	
	mean	95% CI	mean	95% CI	mean	95% CI	mean	95% CI	mean	95% CI
The whole cohort	6.98	6.12 7.84	1.15	1.08 1.22	26.4	22.9 29.9	17.2	16.6 17.7	−0.27	−0.55 0.02
*n = 155*	*155*		*117*		*118*		*117*		*117*	
< 2 years	1.07	0.90 1.24	0.71	0.68 0.75	8.6	7.7 9.4	16.6	15.6 17.7	−0.31	−0.96 0.33
*n = 41*	*41*		*29*		*29*		*29*		*29*	
2 - < 10 years	5.39	4.80 5.97	1.07	1.02 1.11	18.8	16.8 20.7	16.2	15.4 17.0	−0.29	−0.79 0.22
*n = 64*	*64*		*52*		*52*		*52*		*52*	
10–19 years	13.87	13.26 14.49	1.62	1.57 1.66	51.1	47.1 55.1	19.1	18.2 20.0	−0.20	−0.54 0.12
*n = 50*	*50*		*36*		*37*		*36*		*36*	

BMI: body mass index

### FAs and indices

Table 2 shows the analysed FAs and indices in the three age groups. Of the 21 FAs and indices analysed, 14 changed significantly according to age. Significant differences were observed between the group of small children and the two groups of subjects 2 years and older. An exception was FADS1 that differed between the group of small children and the school age group only.

**Table 2:** Whole blood fatty acids and indices in subjects aged 46 days to 19 years without acute, chronic, or inflammatory disease

	< 2 years (*n* = 40)			2- < 10 years (*n* = 62)			10–19 years (*n* = 50)		
Fatty acids (%)	mean	median	95% CI	mean	median	95% CI	mean	median	95% CI
16:0	23.32	23.52	22.64 24.01	22.81	22.63	22.44 23.18	23.19	23.01	22.78 23.60
**18:0**	11.35	11.45	10.90 11.80	12.10^a^	12.02	11.80 12.41	12.26^b^	12.27	11.93 12.59
**18:1n9**	21.88	21.72	20.93 22.83	19.88^c^	19.49	19.09 20.68	18.88^c^	18.20	18.09 19.67
LA	20.36	20.29	19.57 21.15	21.14	21.00	20.44 21.84	20.86	21.10	20.19 21.54
**DGLA**	1.23	1.25	1.14 1.32	1.58^c^	1.59	1.50 1.65	1.54^c^	1.54	1.46 1.63
**AA**	8.22	8.41	7.69 8.76	9.59^c^	9.78	9.20 9.98	9.82^c^	9.92	9.48 10.16
**ALA**	0.47	0.41	0.36 0.58	0.21^c^	0.19	0.19 0.24	0.19^c^	0.17	0.17 0.21
EPA	0.30	0.29	0.25 0.36	0.31	0.27	0.26 0.36	0.28	0.26	0.24 0.35
DHA	2.07	2.03	1.82 2.33	1.85	1.78	1.69 2.01	1.85	1.82	1.69 2.02
Total SFA	38.91	38.70	37.79 40.02	38.82	38.64	38.22 39.41	39.48	39.39	38.80 40.16
**Total MUFA**	26.30	26.39	25.36 27.24	23.92^c^	23.54	23.12 24.72	23.30^c^	22.73	22.53 24.08
**Total PUFA**	34.80	34.98	33.71 35.88	37.26^b^	37.49	36.34 38.19	37.22^b^	37.21	36.33 38.11
**Total n6-PUFA**	31.26	31.70	30.26 32.26	34.19^c^	34.64	33.29 35.09	34.21^c^	33.97	33.41 35.01
**Total n3-PUFA**	3.45	3.50	3.13 3.77	2.98^a^	2.91	2.76 3.20	2.91^a^	2.89	2.69 3.13
*Indices*									
PUFA balance^1^	6.79	6.46	6.04 7.54	5.81	5.52	5.29 6.32	5.70	5.49	5.24 6.17
**SCD(18)**^2^	1.98	1.83	1.82 2.14	1.66^b^	1.60	1.57 1.75	1.56^c^	1.53	1.47 1.66
**FADS2**^3^	6.18	6.56	5.63 6.72	7.57^c^	7.74	7.16 7.98	7.52^b^	7.30	7.02 8.02
**FADS1**^4^	6.91	6.73	6.39 7.44	6.19^a^	6.15	5.90 6.48	6.50	6.52	6.20 6.80
**AA/DHA**	4.51	4.19	3.95 5.07	5.67^b^	5.68	5.23 6.11	5.82^b^	5.39	5.25 6.39
AA/EPA	34.90	30.21	28.33 41.46	41.53	34.79	34.88 48.19	42.86	38.32	36.77 48.95
**n6/n3**	9.89	9.14	8.90 10.88	12.43^b^	12.31	11.48 13.39	12.58^b^	11.87	11.58 13.58

^1^[(%EPA + %DHA)/%Total PUFA] × 100; ^2^(18:1n9/18:0); ^3^(DGLA/LA) × 100; ^4^AA/DGLA; **Bold**: significant differences between groups (Kruskal Wallis test); ^a,b,c^*P* < .05, <.01, <.001 vs age group < 2 years (Bonferroni post hoc analysis). Abbreviations: CI, confidence interval; LA: linoleic acid; DGLA: dihomo-gamma-linolenic acid; AA: arachidonic acid; ALA: alpha-linolenic acid; EPA: eicosapentaenoic acid; DHA: docosahexaenoic acid; SFA: saturated fatty acids; MUFA: monounsaturated fatty acids; PUFA: polyunsaturated fatty acids; n6: omega-6; n3: omega-3; SCD: stearoyl-CoA desaturase; FADS: fatty acid desaturase

### Dietary data

Dietary information was obtained from 60 subjects. The information obtained during the interview and the 24-h dietary recall was extracted into 15 questions in the KIDMED questionnaire (Table 3).

**Table 3:** The KIDMED questionnaire

	If yes
1. Consumption of a piece of fruit or fruit juice every day?	+ 1
2. Consumption of a second fruit every day?	+ 1
3. Consumption of fresh or cooked vegetables once a day?	+ 1
4. Consumption of fresh or cooked vegetables more than once a day?	+ 1
5^a^. Did you consume fish yesterday?	+ 1
6^a^. Did you consume processed or red meat yesterday?	- 1
7. Consumption of pasta or rice once a day?	+ 1
8. Habitually use of olive oil at home?	+ 1
9. Cereals, bread, or rusks at breakfast?	+ 1
10. Skips breakfast?	- 1
11. Consumption of milk or dairy products for breakfast?	+ 1
12. Consumption of snacks or biscuits for breakfast?	- 1
13. Consumption of 2 yoghurts and/or cheese (40 g)/day?	+ 1
14. Consumption of sweets and/or candy several times a day?	- 1
15^a^. Consumption of 5 meals (including 2 snacks) /day?	+ 1

^a^modified question

**Fig. 2:**
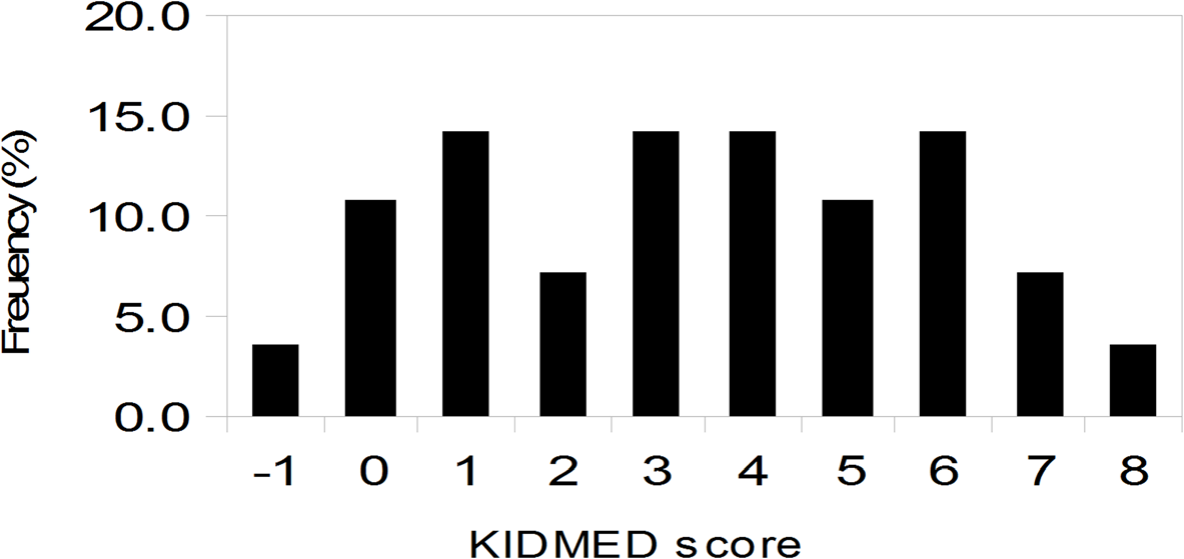
KIDMED score distribution in children aged 2- < 10 years (*n* = 28)

The results showed that 8.3% of the participants obtained a score indicative of a high adherence to the MedDiet and 91.7% showed a low-intermediate adherence. The group of small children included subjects that received breast milk or milk in formula at the time of blood sampling and were excluded from further considerations on diet. The adolescent group was not further investigated either. Regarding the school-age group of children, 89.3% showed a low-intermediate adherence level to the MedDiet (Fig. 2).

### Correlation KIDMED score and select FAs and indices in school age children

Dietary data was available for 28 school age children, of which two lacked FA data. The KIDMED score correlated positively with total MUFA and PUFA balance, and negatively with total PUFA, total n6-PUFA, and the ratios AA/DHA, AA/EPA, and n6/n3 (Table 4). Adjusting for age, the score correlated with PUFA balance and the AA/EPA ratio (Table 4). The score did not correlate with oleic acid, LA, AA, ALA, EPA, DHA, and total n3-PUFA (Table 4) nor with DGLA, total SFA, FADS2 and FADS1 (data not shown).

**Table 4:** Correlation between KIDMED score and fatty acids and indices in children 2- < 10 years old (*n* = 28)

Fatty acid, indices	Correlation coefficient	Correlation controlled for age
18:1n9	.476	.261
LA	−.515	−.296
AA	−.287	−.111
ALA	.415	.331
EPA	.451	.453
DHA	.256	.352
Total MUFA	.564^a^	.365
Total PUFA	-.631^a^	−.425
Total n6-PUFA	-.678^b^	−.511
Total n3-PUFA	.322	.350
PUFA balance	.548^a^	.531^a^
SCD(18)	.492	.271
AA/DHA	-.554^a^	−.508
AA/EPA	-.625^a^	-.566^a^
n6/n3	-.593^a^	−.511

Statistical significance was evaluated with Spearman’s correlation coefficient. Adjustment for age was done using Spearman’s partial correlation coefficient. ^a^, ^b^, *P* < .01, .001

## Discussion

The present work was performed in a single centre and it describes the FA composition in spotted whole blood in a cohort of young people, their adherence to the MedDiet as evaluated in the KIDMED test, and the correlation between the KIDMED score and various FAs and FA indices.

The majority of the subjects were of male sex. All age groups showed lower BMI and z-BMI values. In fact, the cohort was characterized by being less afflicted by overweight and obesity (10%) than the national (29.7%) or regional (22.3%) average in 8-year-olds [22].

As to the FA pattern, some n6-PUFA increased from age group < 2 years to the age groups ≥2 years (DGLA, AA, total n6-PUFA) while LA was stable. There was a trend of an increase in AA levels between school age children and teenagers. The n3-PUFA ALA and total n3-PUFA decreased, while EPA and DHA were stable. Total PUFA was also stable. Among saturated FAs, SA increased while PA and total SFA were invariant.

The FA profile in several blood compartments has been studied for decades as marker for dietary fat intake [23]. Besides dietary intake of fat, further factors may influence the FA profile of tissues such as repletion of vitamins and trace elements which was shown to improve children’s circulating LCPUFA status [24, 25]. Also exposure to environmental pollutants may affect FA composition: urinary phthalates correlated with circulating FAs in the general adult US population, and various phthalates correlated with distinct FAs [26]. A recent report on global Bisphenol A intake showed that Italian children had the lowest daily exposure dose among children from 18 regions/countries [27], although children living in Milan were shown to be exposed to environmental equivalent black carbon which depended on season and further variables [28]. The genetic make-up also seems to be a major factor in determining FA levels as well, depending on FADS variants [29–31]. Globally, the levels revealed here should reflect the impact of all variables that influence blood fatty acids.

To evaluate compliance to the MedDiet, a modified version of the KIDMED protocol was used. The questions on pulses and nuts were not detected in the 24-h recall and these queries were omitted. The consumption of fish and meat was traced and the questions about these foods were transformed into consumption thereof on the day before the interview, and the item ‘fast food’ was rephrased to ‘transformed/red meat’. Also, the topic of the daily distribution of meals is addressed in the Italian guidelines in relation to healthy eating [32] and a question in that regard was introduced in the questionnaire. The changes might have made the survey less stringent, but several questionnaires of KIDMED exist [21] and adapted protocols are used [33–35]. Generally, the cohort showed scarce compliance to the MedDiet: about 10% showed a high level. In the age group 2- < 10 yrs., the MedDiet score correlated significantly with some of the FA parameters and ratios. The score correlated negatively with total PUFA, total n6-PUFA, AA/DHA, AA/EPA, and n6/n3; positively with total MUFA and PUFA balance. The negative effect of the score on blood n6-FA parameters and the related neutral/positive effect on n3-PUFA resulted in a changed equilibrium in blood PUFA which was reflected in the PUFA balance. Age influenced correlations but not those regarding PUFA balance and AA/EPA ratio. There was a strong trend for a negative correlation between the score and LA. There was also a trend for a positive correlation between score and oleic acid. Oleic acid is an important component of olive oil which is maybe the cardinal marker of the MedDiet [36]. Unfortunately, the cohort number with dietary data was small which plausibly may explain the non-significant correlation between score and oleic acid, LA, and some other FAs.

### Comparisons with other studies and what the current work adds to the existing knowledge

Regarding FAs in western children without apparent disease, early and recent studies analysed the FA composition in plasma [37, 38], red blood cells (RBC) [39], and whole blood, as mentioned [13–15]. The FA profile observed in the present study corroborates partly previous findings in young Italian individuals without apparent disease [13]. In that study, the pattern of changes in the FA composition from neonatal age to childhood showed an increase in some FAs (LA, ALA, PUFA, and omega-6), a decrease in others (AA, DHA, SFA, MUFA, omega-3), or unaltered levels (EPA) [13]. The findings noted here regarding total PUFA, n6-PUFA, MUFA, and EPA corroborate those earlier observations whereas those on LA, ALA, AA, DHA, and SFA do not. It may be noted that the infants in the former study were 4 days old neonates whereas here, the youngest child was 46 days. The different ages of the youngest children in the two studies complicate comparisons and may explain differences between the results. The group with older children showed a higher mean BMI than the subjects studied here [13]. It is known that the FA metabolism changes in obesity: the desaturating enzyme activity of FADS2 appeared to increase while that of FADS1 appeared to be inhibited with the result of lower LCPUFA levels [5]. This could explain discrepancies in the results, for instance, in the present study, DHA and AA levels were higher than in the report by Risé et al. [13]. Despite this, the estimated FADS activities detected here overlapped with those seen in other surveys [14, 15]. The “Identification and prevention of dietary-and lifestyle-induced health effects in children and infants” (IDEFICS) project, which aims also included the search for biomarkers of the aetiology of overweight, obesity, and related disorders [40], is the largest survey performed so far on spotted whole blood FA profile in children. IDEFICS presented FA reference intervals from European children 3–9 years [14]. In that survey, a few FAs were weakly age-dependent (LA, EPA, DPAn3 in the girls), some were stable throughout the age range (ALA, DHA, AA). Several investigations described FAs in spotted whole blood from children in developing countries [41–43], some of which showed high levels of EPA and DHA [42, 43].

As mentioned earlier, the FA profile in several blood compartments has been studied as a marker for dietary fat intake [23]. A few studies evaluated the dietary intake and its relation to whole blood FAs in adults [19, 44, 45] and in Danish children [46, 47]. The low compliance to the MedDiet in the present research is in accordance with observations in several countries in southern Europe [17] and Israel [35] and support earlier data from the ZOOM8 study on 8–9 year old children in Italy [33]. Similarly to what could be observed here, a lack of nut consumption was seen by the children in the Calabrian Sierras Community study (CSCS) in Southern Italy [34]. In contrast to the present results though, most of the children in the CSCS study showed high-intermediate adherence to the MedDiet. As far as we are aware, the current study is the first to estimate correlations between the KIDMED test score and the FA profile in whole blood in children. Recently, the KIDMED index was used in the evaluation of FAs in red cell membranes in a study on childhood obesity [48]. In that report, the cohort comprised a higher number of participants than in the present one and weak but significant correlations were revealed between the KIDMED score and AA, EPA, and DHA [48]. Whole blood consists of plasma and cells, mostly RBC [23], and the content of EPA and DHA correlated strongly with that of whole blood [49]. The directions of the correlations detected here in whole blood agreed with those seen in RBC membranes [48]. Lack of significant correlations was noticed for some PUFA, which may be due to the small number of participants already discussed. Various health outcomes in children could be related to whole blood FAs [15, 46, 47] and the current study shows the possibility of using whole blood FAs and the MedDiet score in the study of childhood health issues.

### Strengths and limitations of the study

A strength of this study regards the relatively large cohort of paediatric subjects from a single centre that permitted relatively homogeneous data. Another strength concerns the choice of whole blood for FA measurements that showed its relevance in studying FA and MedDiet relationships. The study has some limitations though, first of all, dietary data was not available from all participants. This probably allowed only the strongest correlations between KIDMED score and FA parameters to reach significance. Further, the use of a modified KIDMED questionnaire may have reduced accuracy, even if it is well known and accepted that a number of adaptations are applied.

## Conclusions

In summary, this single-centre survey reports whole blood FAs and indices in infants, children, and adolescents which add information and knowledge to the existent literature about FA composition in young subjects. Increased FA proportions with age were seen in the n6-series of PUFA. The n3- PUFA species decreased or were stable. The vast majority of the subjects with dietary data, 92%, obtained a KIDMED score indicative of low-medium adherence to the MedDiet. In children 2- < 10 years of age, the score correlated positively with PUFA balance and negatively with various n6-PUFA species. This combination indicates that the MedDiet dampens circulating n6-PUFA. The results show the relevance of using whole blood to study presumed relationships between MedDiet and FAs and could be used in the clinic in studies of childhood health problems.

## Data Availability

Data generated during the study will be available upon reasonable request to the corresponding author.

## Abbreviations

FAs: fatty acids
PUFA: polyunsaturated fatty acids
SFA: saturated fatty acids
MUFA: monounsaturated fatty acids
LCPUFA: long chain polyunsaturated fatty acids
DGLA: dihomo-gamma-linoleic acid
AA: arachidonic acid
EPA: eicosapentaenoic acid
DHA: docosahexaenoic acid
LA: linoleic acid
ALA: alpha-linolenic acid
MedDiet: mediterranean diet
KIDMED: the Mediterranean Diet Quality Index for Children
RBC: red blood cell
